# Anti-Inflammatory Activity of Three Triterpene from *Hippophae rhamnoides* L. in Lipopolysaccharide-Stimulated RAW264.7 Cells

**DOI:** 10.3390/ijms222112009

**Published:** 2021-11-05

**Authors:** Yu Han, Chen Yuan, Xiaowei Zhou, Yingjie Han, Yanhao He, Jian Ouyang, Wenna Zhou, Zhenhua Wang, Honglun Wang, Gang Li

**Affiliations:** 1 Center for Mitochondria and Healthy Aging, College of Life Sciences, Yantai University, Yantai 264005, China; hanyu912231674@163.com (Y.H.); ytu_yc@163.com (C.Y.); xiaoweiz1223@163.com (X.Z.); 17853509381@163.com (Y.H.); ythyh@ytu.edu.cn (Y.H.); skywzh@ytu.edu.cn (Z.W.); 2 Key Laboratory of Tibetan Medicine Research, Northwest Institute of Plateau Biology, Chinese Academy of Sciences, Xining 810008, China; ygzjj@126.com (J.O.); hlwang@nwipb.cas.cn (H.W.); 3 Department of Life Sciences and Health, QiuZhen College, Huzhou University, Huzhou 313000, China; 02638@zjhu.edu.cn

**Keywords:** inflammation, RAW264.7 cell, oleanolic acid, asiatic acid, maslinic acid, NF-κB

## Abstract

Oleanolic acid (OA), asiatic acid (AA), and maslinic acid (MA) are ubiquitous isomeric triterpene phytochemicals with many pharmacological effects. To improve their application value, we used lipopolysaccharide (LPS) to induce RAW264.7 cells and studied the differences in the anti-inflammatory effects of the triterpenes according to their structural differences. MTT, Griess, and immunofluorescence assays, ELISA, flow cytometry, and Western blotting, were performed. The release of LPS-induced pro-inflammatory mediators, such as nitric oxide (NO), inducible nitric oxide synthase (iNOS), and interleukin (IL-6), was significantly inhibited by OA, AA, and MA at the same concentration, and AA and MA promoted the production of anti-inflammatory factor IL-10. OA, AA, and MA inhibited LPS-induced NF-κB nuclear translocation in RAW264.7 cells. OA and AA inhibited the phosphorylation of ERK1/2, P38, and JNK1/2 in LPS-stimulated RAW264.7 cells. Moreover, OA increased LPS-induced Nrf2 expression and decreased Keap1 expression in RAW264.7 cells. OA, AA, and MA inhibited LPS-stimulated intracellular reactive oxygen species (ROS) production and alleviated mitochondrial membrane potential depletion. Overall, our data suggested that OA, AA, and MA exhibited significant anti-inflammatory effects in vitro. In particular, OA and AA take effects through the MAPKs, NF-κB, and Nrf2 signaling pathways.

## 1. Introduction

Inflammation involves a series of abnormal pathological reactions in the body caused by the invasion of pathogenic microorganisms. The entire process is strictly regulated by inflammation-related signaling molecules, which are closely associated with the occurrence, maintenance, and regression of inflammation [1]. Thus, screening anti-inflammatory compounds based on signaling molecules involved in the inflammation signaling pathways is an important approach. Currently, nonsteroidal and steroidal anti-inflammatory drugs are commonly used in clinical practice. However, long-term and frequent use may lead to adverse reactions. Natural medicines have the advantages of availability from a wide range of sources, low cost, and few side effects. The anti-inflammatory effect of some natural medicines has been recognized by increasing research [2].

*Hippophae rhamnoides* L. is a shrub or small tree, rich in vitamins, carotene, triterpenoids, glycosides, flavonoids, alkaloids, and other bioactive substances with antioxidant, immune-regulating, antistress, anti-inflammatory, and other biological functions [3,4]. Studies have shown that its fruit is rich in maslinic acid (MA), oleanolic acid (OA), and ursolic acid [4]. Triterpenoids are compounds in which the basic parent nucleus is composed of 30 carbon atoms. Triterpenoids exist in plants either in free form or in the form of glycosides or esters combined with sugars and are known to have various biochemical activities. Terpenes are widely distributed in nature and many derivatives inhibit tumor progression, inflammation, and lipid peroxidation. The anti-inflammatory activity of the triterpenoid acids OA, asiatic acid (AA), and MA has been separately demonstrated [5,6,7]. Oleanolic acid is an oleanane-type five-ring triterpenoid compound. It is the main functional component of several natural products and exists in a variety of plants in the free or glycoside forms. Current studies have found that in addition to anti-inflammatory, hypoglycemic, immune-enhancement, antioxidant, platelet aggregation inhibition, and diuretic effects, OA also has hepatoprotective, anti-atherosclerotic, anti-hyperlipidemic, and other clinical pharmacological effects. The development prospects of medicinal use are broad owing to its antitumor and other biological activities [8]. AA is pentacyclic triterpene acid present in many plants. AA content is relatively high in *Centella asiatica*, which is the main source of AA. It has been used as a medicinal plant in China for over 2000 years and is a commonly used Chinese medicine included in the Chinese Pharmacopoeia. *C. asiatica* resources are abundant in China, especially in the South of the Yangtze River basin. Studies have shown that AA has anti-inflammatory, skin-repairing, hepatoprotective and lung-protective, antidepressant, hypoglycemic, hypolipidemic, neuroprotective, and antitumor effects [9,10,11]. MA is a pentacyclic triterpenoid acid widely found in loquat leaves, olives, maslinic, and other plants [12,13]. In recent years, its antitumor [12], hypoglycemic [14], anti-inflammatory [15], antiparasitic [16], and other biological activities have been demonstrated, indicating its potential as a therapeutic drug.

However, to the best of our knowledge, the differences in anti-inflammatory activity and the mechanism of action of these three compounds have not been reported. Therefore, in this study, we compared the sites of action and mechanistic trends of these three compounds by determining the relevant inflammatory indicators.

The anti-inflammatory activity of MA and OA isolated from *Hippophae rhamnoides* L. by the Northwest Institute of Plateau Biology was investigated. Previous studies have shown that AA is a triterpenoid isomer with anti-inflammatory activity [5].

**Figure 1:**
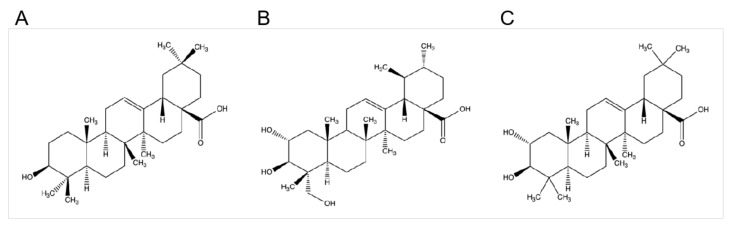
Chemical structures of oleanolic acid (OA) (**A**), asiatic acid (AA) (**B**), and maslinic acid (MA) (**C**).

In this study, we investigated the potential role of OA, AA, and MA (Figure 1) on inflammation induced by LPS in RAW264.7 cells. The mechanism by which OA, AA, and MA exerted their effects on the inflammatory response has been discussed.

## 2. Results

### 2.1. Cytotoxicity of OA, AA, and MA

**Figure 2:**
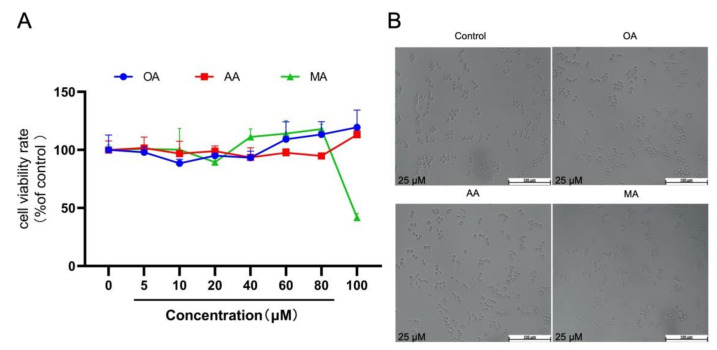
Effect of OA, AA, and MA on cell viability (**A**) RAW264.7 cells were treated with different concentrations of compounds (0–100 μM) for 24 h. Cell viability was analyzed using the MTT assay. (**B**) Cells were treated with 25 μM OA, AA, and MA for 24 h.

The 3-(4,5-dimethylthlthiazol-2-yl)-2,5-diphenyltetrazolium bromide (MTT) assay was used to determine the cytotoxicity of OA, AA, and MA in RAW264.7 cells. As shown in Figure 2A, OA and AA (5–100 μM) had no significant effect on RAW264.7 cell viability compared with the controls, whereas a high concentration (100 μM) of MA significantly inhibited RAW264.7 cell viability. The cells appeared round and bright at OA, AA, and MA concentrations of 25 μM, and there was no obvious enlargement or stretching. Microscopy revealed no significant changes in cell morphology (Figure 2B). Therefore, the three compounds were used at a concentration of 25 μM in subsequent experiments.

### 2.2. Effects of OA, AA, and MA on the Production of Nitric Oxide (NO), Inducible Nitric Oxide Synthase (iNOS) and Inflammatory Factors in LPS-Stimulated RAW264.7 Cells

**Figure 3:**
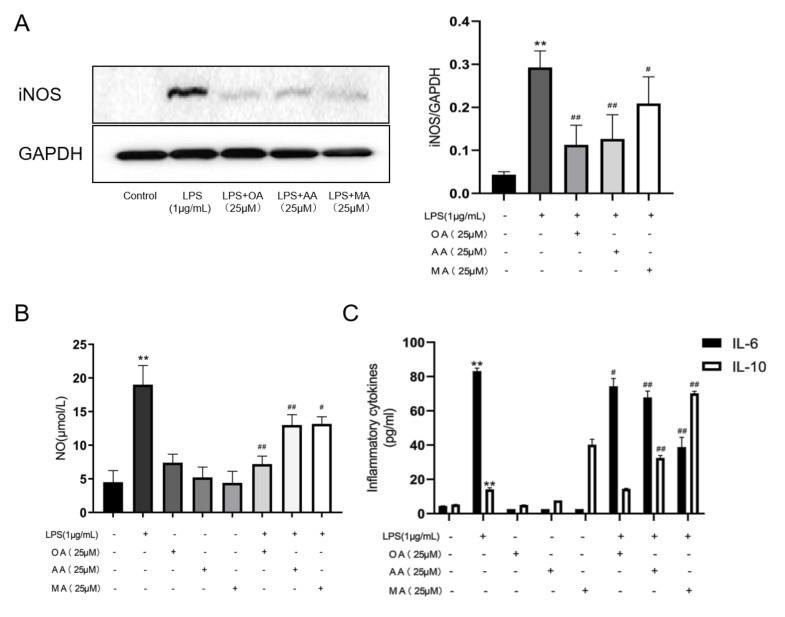
Effects of OA, AA, and MA on the production of NO, iNOS, and inflammatory factors in LPS-stimulated RAW264.7 cells. (**A**) Macrophages were treated with 25 μM OA, AA, and MA (with or without LPS) for 10 h to detect iNOS expression in cells. (**B**) Macrophages were treated with 25 μM OA, AA, and MA (with or without LPS) for 24 h, and NO content in the cell supernatants was measured. (**C**) Macrophages were treated with 25 μM OA, AA, and MA (with or without LPS) for 24 h and IL-6 and IL-10 were detected using ELISA. Each bar represents the mean ± standard deviation (SD) of three set of measurements from three different experiments. ** *p* < 0.01 vs. control group; ^#^ *p* < 0.05, ^##^ *p* < 0.01 vs. LPS-treated group.

When macrophages are stimulated and activated, a large amount of NO is released to kill the pathogenic microorganisms in the body and induce an inflammatory response to resist the invasion of adverse external factors. Therefore, in this study, we investigated whether the three compounds affected NO production in LPS-induced RAW264.7 cells. NO levels in the cell supernatant were determined using Griess reagent. The results indicated that the NO content in RAW264.7 cells stimulated by LPS was significantly increased compared with that in the control group. Compared with that in the model group, OA had the strongest inhibitory effect on NO production in RAW264.7 cells stimulated by LPS followed by AA and MA. In general, the three compounds reduced NO content in macrophages to different degrees (Figure 3B).

The inducible isoform iNOS is involved in the immune response and produces NO, an important cell-signaling molecule. NO expression is controlled by iNOS, a key upstream enzyme. Therefore, we investigated whether the three compounds affected iNOS expression in LPS-stimulated RAW264.7 cells. Compared with that in the control group, iNOS protein expression in RAW264.7 cells was significantly increased under LPS stimulation. Compared with LPS alone, OA had the strongest inhibitory effect on the iNOS production in LPS-induced RAW264.7 cells, followed by AA and MA. In general, these three compounds lowered iNOS expression in RAW264.7 cells after to varying degrees after LPS induction (Figure 3A).

RAW264.7 cells activated under different stimuli have different functions. The first is a pro-inflammatory M1 (typically activated macrophage) function and the second is an anti-inflammatory M2 (alternatingly activated macrophage) function. M1 macrophages produce high levels of pro-inflammatory mediators including IL-1β, IL-6, IL-12, and tumor necrosis factor (TNF)-α, whereas M2 macrophages have glycogen receptors (MRC1/CD206) and produce high levels of anti-inflammatory mediators such as arginase-1 (Arg1) and IL-10. Therefore, we investigated whether the three compounds affected the production of inflammatory cytokines in LPS-induced RAW264.7 cells. IL-6 and IL-10 levels were determined using enzyme-linked immunosorbent assay (ELISA). The results showed that the IL-6 and IL-10 levels in the supernatant of LPS-induced RAW 264.7 cells were significantly increased (*p* < 0.01). Compared with that in the LPS-treated group, OA, AA, and MA treatment inhibited IL-6 release in the cell supernatant and the inhibitory effect of MA was the strongest (Figure 3C). Furthermore, AA and MA promoted IL-10 release in LPS-stimulated macrophages and MA had a stronger promoting effect (Figure 3C).

### 2.3. OA, AA, and MA Reduce ROS Content and Restore Mitochondrial Membrane Potential in LPS-Stimulated Macrophages to Varying Degrees

**Figure 4:**
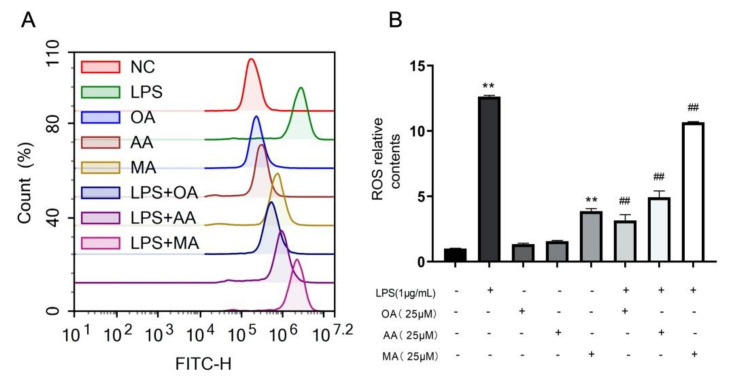
Effects of OA, AA, and MA on ROS content. (**A**) Macrophages were incubated with OA, AA, and MA at 25 μM for 10 h (with or without LPS). DCFH2-DA staining was performed for 30 min. Fluorescence intensity was detected using flow cytometry. (**B**) Data analysis of ROS production in each group. All data are expressed as the mean ± SD. ** p < 0.01 vs. control group. *^##^ p* < 0.01 vs. LPS-treated group.

Reactive oxygen species (ROS) are very important signal transduction factors playing an important role in certain inflammatory diseases. LPS can induce increases in ROS in RAW264.7 cells. Inflammation can also increase ROS levels, further promoting oxidative stress response in cells. DCFH2-DA was used in this experiment to determine ROS production. As shown in Figure 4, ROS content was significantly increased after LPS induction in RAW264.7 cells compared with the control. Compared with that in the model group, OA, AA, and MA treatment significantly inhibited LPS-induced ROS production in macrophages, with OA having the strongest effect in reducing intracellular ROS levels, followed by AA and MA (Figure 4).

**Figure 5:**
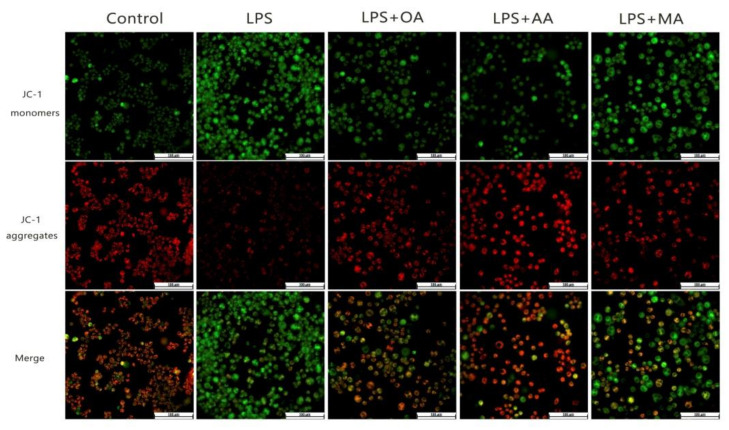
OA, AA, and MA decreased the mitochondrial membrane potential (MMP) of LPS-induced macrophages. Macrophages were incubated with OA, AA, and MA at 25 μM for 10 h (with or without LPS). The RAW264.7 cells were incubated with the JC-1 (10 μM) probe for 30 min and images were acquired using fluorescence microscopy. Scale bar = 100 µM.

The mitochondrial membrane potential (MMP) is altered after LPS induction and has a certain adverse effect in maintaining the normal physiological functions of cells. As shown, when RAW264.7 cells were activated, JC-1 monomers increased and remained in a free state (green staining), whereas after pretreatment with OA, AA, and MA, JC-1 monomers decreased and resumed aggregation (red fluorescence). Moreover, AA showed the best effect on MMP recovery, followed by OA and MA (Figure 5). In conclusion, OA, AA, and MA inhibited ROS production to different degrees and enhanced the MMP in RAW264.7 cells.

### 2.4. Effects of OA, AA, and MA on MAPK Pathways in LPS-Stimulated RAW264.7 Cells

**Figure 6:**
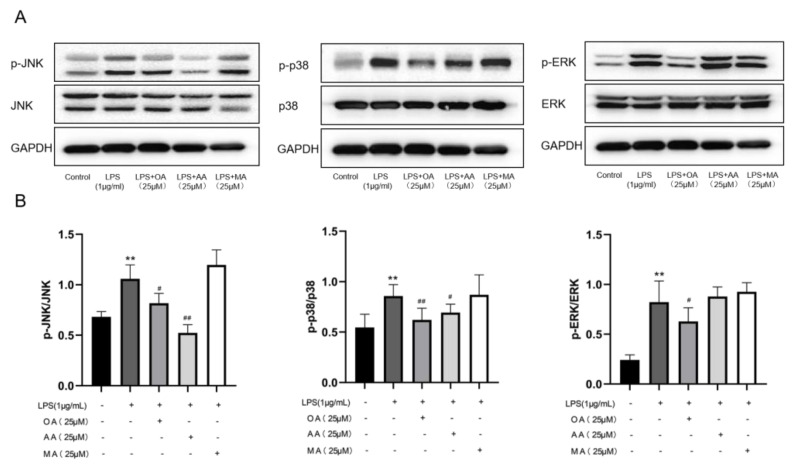
Effects of OA, AA, and MA on the MAPK pathway in macrophages. (**A**) OA, AA, and MA-treated LPS-induced macrophages. Proteins expressed in the MAPK inflammation signaling pathway were detected using Western blotting. (**B**) Proteins of each group were analyzed statistically. All data are displayed as the mean ± SD. *n* = 3. ** p < 0.01 vs. control group. *^#^ p* < 0.05, *^##^ p* < 0.01 vs. LPS-treated group.

LPS can induce and activate the mitogen-activated protein kinase (MAPK) pathway. MAPKs have been found to contain three downstream pathways, extracellular signal-regulated kinase (ERK1/2), Janus kinase (JNK), and p38 [17]. According to Western blotting results in Figure 6, the MAPK signaling pathway was activated after LPS induction of macrophages, and OA significantly reduced the protein expression levels of P-ERK1/2, P-P38MAPK, and P-JNK1/2, but did not affect the total protein expression of JNK1/2, ERK1/2, and p38MAPK. AA significantly inhibited the protein expression of p-JNK1/2 and p-p38MAPK but not the total protein expression of JNK1/2 and p38MAPK. However, MA did not significantly inhibit the protein phosphorylation levels of p38MAPK, ERK1/2, and JNK1/2, and did not affect the total protein expressions of JNK1/2, p38MAPK, and ERK1/2. On the contrary, it could activate the protein expression of p-JNK1/2, p-ERK1/2, and p-p38MAPK to some extent. In summary, the data in this study suggested that in the LPS-stimulated macrophage model, OA regulated MAPK signaling pathway proteins, and AA regulated JNK1/2 and p38MAPK signaling proteins, demonstrating anti-inflammatory activity.

### 2.5. Effects of OA, AA, and MA on the NF-κB Pathway in LPS-Stimulated RAW264.7 Cells

**Figure 7:**
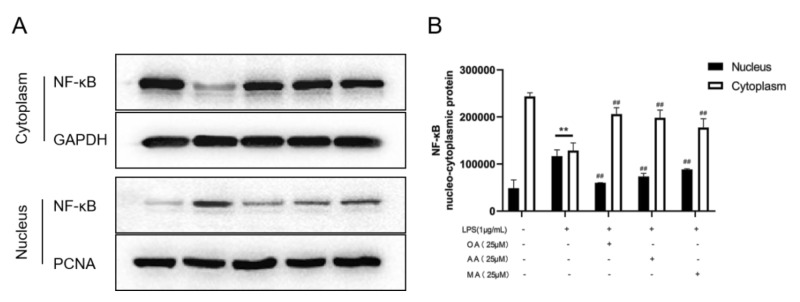
OA, AA, and MA inhibited LPS-stimulated NF-κB translocation in macrophages. Macrophages were treated with 25 μM OA, AA, and MA (with or without LPS) for 10 h. (**A**) Translocation of NF-κB in cells was detected using Western blotting. (**B**) Expression levels of NF-κB in each group were analyzed statistically. All data are presented as the mean ± SD, *n* = 3. ** *p* < 0.01 vs. control group. ^##^ *p* < 0.01 vs. LPS-treated group.

After LPS induction of RAW264.7 cells, NF-κB was transferred from the cytoplasm to the nucleus. NF-κB translocation promotes the release of high levels of inflammatory mediators such as TNF-α, IL-6, and IL-1β [18]. As shown, LPS-induced macrophages promoted NF-κB nuclear transport compared with that in the control group. Compared with that in the model group, OA, AA, and MA treatment (25 μM) exhibited different inhibitory effects on NF-κB translocation (Figure 7), and OA had the strongest inhibitory effect on nuclear translocation. Based on the above results, we concluded that all three compounds could inhibit the nuclear translocation of NF-κB and exhibit different effects.

### 2.6. Effects of OA, AA, and MA on the Keap1-Nrf2 Pathway in LPS-Stimulated RAW264.7 Cells

**Figure 8:**
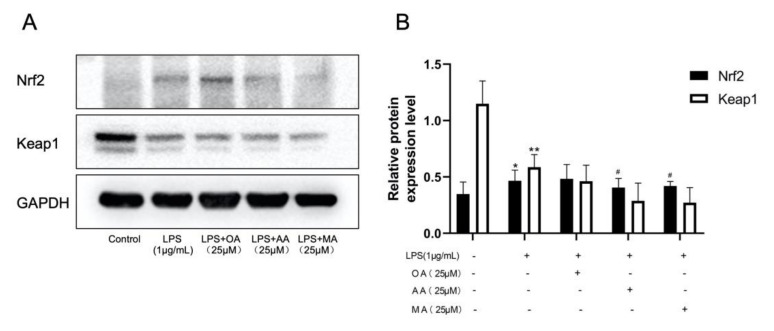
Effects of OA, AA, and MA on the Keap1-Nrf2 pathway in macrophages. (**A**) RAW264.7 cells were induced with LPS and treated with OA, AA, and MA. Western blotting was used to detect Keap1 and Nrf2 protein expression. (**B**) Expression levels of Nrf2 and Keap1 in each group were analyzed statistically. Groups of gels/blots cut from the same gel or from different parts of a different gel. All data are presented as mean ± SD, *n* = 3. * *p* < 0.05, ** *p* < 0.01 vs. control group. ^#^ *p* < 0.05 vs. LPS-treated group.

Nrf2 is the main protein that regulates the expression of heme oxygenase (HO)-1, Keap1 is distributed in the cytoplasm. After cell stimulation, Keap1 dissociates from Nrf2 and degrades under oxidative stress, whereas Nrf2 dissociates and is transferred into the nucleus. Our findings revealed that LPS-induced macrophages promoted Nrf2 nuclear transport, Nrf2 translocation, and Keap1 degradation. As shown in Figure 8, AA and MA inhibited Nrf2 release and at the same time, inhibited the separation of Keap1 and Nrf2.

## 3. Discussion

Inflammation is a defense or immune response of the body to external stimulus, but excessive or persistent inflammation can cause several inflammation-related diseases [19]. As a pattern-recognition molecule, LPS plays an important role in the study of the mechanisms of natural immunity. Previous studies have shown that the cascade reaction of inflammatory factors, such as macrophages induced by LPS, causes much more damage to tissue cells than its own influence on the body [20]. After induction by LPS, macrophages not only significantly increase cell proliferation and phagocytosis but also release high levels of inflammatory cytokines and inflammatory mediators such as NO, TNF-α, IL-1β, and IL-6. These mediators can resist the invasion of pathogenic microorganisms, and excessive secretions result in inflammatory reactions [21,22]. Therefore, we used LPS to establish a RAW264.7 cell-inflammation model. 

In our earlier study, we found that OA, AA, and MA had small structural differences, but each compound had a certain extent of anti-inflammatory activity and could reduce inflammation. LPS can bind to the toll-like receptor (TLR)4 receptor on the surfaces of cell membranes and promote information transmission [23]. We used LPS to establish a RAW264.7 model of cell inflammation. Macrophages induced by LPS can release many inflammatory mediators, such as NO, IL-6, and IL-10 [24]. Therefore, factors affecting the inflammatory response may affect the transmission of information during inflammation. In our study, OA, AA, and MA reduced the production of NO, iNOS, and IL-6 at the same concentration without obvious toxicity to RAW264.7 cells. AA and MA significantly increased the levels of the anti-inflammatory cytokine IL-10. These results showed that all three compounds had different anti-inflammatory activities.

Several inflammatory signaling pathways, such as the MAPK, NF-κB, and Nrf2 pathways are activated after the stimulation of macrophages by LPS [20]. NF-κB is a nuclear transcription factor. Under normal circumstances, NF-κB is in an inactive state due to the inhibitory effect of IκB. When cells are stimulated by LPS, IκB will be phosphorylated when activated by the IKKα and IKKβ complex, so that NF-κB will no longer be inhibited. At this time, NF-κB is separated from IκB, leading to NF-κB activation and its transfer to the nucleus, inducing the activation and expression of TNF-α, IL-1, and other immunoinflammatory mediators [25,26]. Li et al. found that OA could inhibit nuclear NF-κB metastasis in fibroblast-like synovial cell inflammation induced by TNF-α [27]. Chen Si-yun et al. have reported the NF-κB signaling pathway to be one of the important regulatory pathways in exploring the mechanism of AA in alcoholic hepatitis [28]. In addition, MA was shown to inhibit the NF-κB pathway in studies exploring the mechanisms by which MA reduced the inflammatory response and oxidative stress levels in mice with acute liver injury [29]. In this study, our findings revealed that OA, AA, and MA inhibited the nuclear translocation of NF-κB to different degrees and that OA had the strongest inhibitory effect. MAPK signaling pathway is involved in the inflammatory response [30]. We found that LPS-induced RAW264.7 cells increased the phosphorylation of P38, ERK1/2, and JNK1/2. OA significantly inhibited LPS-induced phosphorylation of P38, JNK1/2, and ERK1/2, and AA significantly reduced the phosphorylation of JNK1/2 and P38 but did not change the total protein expression levels of JNK1/2, P38, and ERK1/2. Collectively, OA and AA mediated the NF-κB and MAPK pathways, whereas MA mediated the NF-κB pathway. All three triterpenes had anti-inflammatory biological activity, but their regulation methods were different.

ROS is a mediator of inflammation and plays a very important role in inflammation-related diseases. Previous studies have shown that LPS stimulated RAW264.7 cells to increase the production of intracellular ROS. ROS is an upstream signaling molecule that mediates the activation of the NF-κB, MAPK, and other signaling pathways to induce inflammation [31,32]. Studies have reported that mitochondrial dysfunction is closely related to inflammation and that it leads to a series of other metabolic diseases. Therefore, maintaining the normal physiological state of mitochondria is a prerequisite for the normal functioning of the body. Normal MMP is necessary to maintain mitochondrial oxidative phosphorylation and maintain mitochondrial function. Therefore, MMP and ROS are both very important signals of the inflammatory response. Studies have suggested that ROS inhibition and MMP recovery may be therapeutic strategies in managing inflammatory diseases [33]. Our findings showed that OA, AA, and MA significantly reduced ROS production and promoted MMP recovery. The Nrf2-Keap1 signaling pathway plays a role in the mechanism of cellular resistance to endogenous or exogenous oxidative stress and is an important component of the cellular defense system. Studies have shown that ROS production can activate Nrf2, which in turn can reduce ROS-induced oxidative damage by inducing the expression of HO-1 protein [34]. It has also been reported that Nrf2 can inhibit inflammation and reduce tissue damage by inhibiting the activation of the NF-κB pathway [35]. Our results showed that treatment with OA, AA, and MA decreased Keap1 expression in RAW264.7 cells and promoted Nrf2 transfer to the nucleus. In general, all three compounds could reduce inflammation through antioxidation.

There are many causes of inflammation and several studies have found that mitochondrial dysfunction is closely related to inflammation [36,37]. Mitochondria are the energy supply center of the body. Impaired mitochondria function leads to a series of metabolic diseases. Our study explored the function of three compounds to improve mitochondrial damage under inflammatory response. Further studies will be undertaken to determine whether mitochondrial function can be improved by modulating the mitochondrial biogenesis pathway.

## 4. Materials and Methods

### 4.1. Chemicals

OA, AA, and MA (purity > 99%) were provided by the Northwest Institute of Plateau Biology, Chinese Academy of Science. RPMI 1640 medium, fetal bovine serum (FBS), phosphate-buffered saline (PBS), trypsin, penicillin 3-(4,5-Dimethylthiazol-2-yl)-2,5-diphenyltetrazolium bromide (MTT) and the Pierce BCA protein detection kit were obtained from Beijing Biodee Biotechnology Co., LTD (Beijing, China). Antibodies specific for iNOS (#13120), NF-ĸB (#8242), Nrf2 (#12721), Keap1 (#8047), ERK1/2 (#4695), p-ERK1/2 (#4370), JNK1/2 (#9252;), p-JNK1/2 (#4668), p38 (#8690), p-p38 (#4511), and GAPDH (#5174) were purchased from Cell Signaling Technology (Beverly, MA, USA). Horseradish peroxidase (HRP)-conjugated goat anti-mouse antibody and HRP-conjugated goat anti-rabbit antibody were purchased from Cell Signaling Technology. All other chemicals were of reagent grade.

### 4.2. Cell Culture

The murine macrophage RAW 264.7 cell line was purchased from the Cell Bank of Chinese Academy of Sciences (Shanghai, China). RAW 264.7 cells were cultured in RPMI 1640 medium supplemented with 1% penicillin/streptomycin and 10% FBS and incubated in an atmosphere of 5% CO_2_ at 37 °C and subcultured every 2 days.

### 4.3. Cytotoxicity Assay

The growth-inhibiting activity of the three compounds on RAW264.7 cells was determined using the MTT method. Macrophages were inoculated into 96-well microplates with 10^4^ cells in each well for 24 h. Next, the cells were treated with different concentrations of triterpenoid acid (0, 5, 10, 20, 40, 60, 80, and 100 μM) for 24 h. MTT (5 mg/mL) was added to each well for 3 h. The medium was removed and dissolved in DMSO (100 μL/well) with crystal violet. The OD was measured at 490 nm using a microplate reader (Molecular Devices, Sunnyvale, CA, USA).

### 4.4. NO Measurement

Cells were plated at a density of 10^5^ cells/well in 96-well plates and incubated with or without LPS (1 µg/mL) in the absence or presence of the same concentration of OA, AA, and MA (25 µM) for 24 h. The NO content in the culture medium supernatant in each well was determined using the Griess method according to the NO kit instructions. The absorbance was measured at 540 nm using a microplate reader. Fresh culture media was used as blanks for all experiments.

### 4.5. IL-6 and IL-10 Measurement

IL-6 and IL-10 levels were determined using ELISA kits. Cells were cultured in 96-well plates at a density of 10^6^ cells/mL and incubated with 25 μM of the three triterpenoid acids, respectively, for 24 h with or without LPS (1 μg/mL). Cell-free supernatants were collected for the determination of IL-6 and IL-10 concentrations according to the manufacturers’ instructions.

### 4.6. Flow Cytometry

Macrophages were seeded into a 24-well plate at a density of 4 × 10^5^ cells/mL and cultured for 24 h. Then, they were incubated for 10 h with 25 μM of the compounds with or without LPS (1 μg/mL) and further incubated with DCFH-DA (10 μM) for 30min. After incubation, the cells were collected, and the fluorescence intensity was determined using flow cytometry (Hangzhou Aisen Company, Hangzhou, China).

### 4.7. Fluorescence Assay

RAW264.7 cells were cultured on coverglass in 24-well plates at a density of 4 × 10^5^ cells/mL for 24 h. Then, they cells were treated with the 25 μM of the test compounds with or without LPS (1 μg/mL) for 10 h and stained with JC-1 (10 μM) for 30 min. Fluorescence microscopy was used to capture fluorescence images.

### 4.8. Western Blotting

RAW264.7 cells were cultured in 6-well plates at a density of 4 × 10^5^ cells/mL incubated with the same concentration (25 μM) of the three triterpenoid acids for 10 h with or without LPS (1 μg/mL). After incubation, the cells were washed twice with PBS and RIPA cell lysate was added. After lysis on ice, the cells were collected and centrifuged to obtain proteins. A BCA kit was used to measure the protein concentration according to the manufacturer’s instructions. PBS was added to adjust the volume of each sample to the same protein concentration. Loading buffer was added and thoroughly mixed to denature the sample. The samples were separated using 6% or 10% sodium dodecyl sulfate (SDS)-polyacrylamide gel electrophoresis (PAGE) and then transferred to polyvinylidene fluoride (PVDF) membranes. The PVDF membrane was blocked with 5% skim milk for 1 h and reacted overnight at 4 °C with primary antibodies diluted 1:1000. The membrane was washed three times with TBST, incubated at room temperature for 1 h with the secondary antibody (1:500) and washed. The membrane was developed using the Tanon5500 gel imaging system. GAPDH was used as the internal reference protein.

### 4.9. Statistical Analysis

All experimental results were obtained from three independent repeated experiments, and the data are expressed as the mean ± SD. SPSS 18.0 was used for statistical analyses. Differences between groups were compared using unpaired Student’s *t-*test, whereas those between multiple groups were assessed using one-way ANOVA followed by Tukey’s test. A *p*-value of <0.05 was considered statistically significant.

## 5. Conclusions

Our findings confirmed that OA and AA exerted anti-inflammatory effects on macrophages through the NF-κB, MAPK, and Nrf2 signaling pathways, while MA play its role through the NF-κB and Nrf2 signaling pathways. In conclusion, the three compounds have certain inhibitory effects on LPS-induced inflammatory response in vitro and may be used as natural anti-inflammatory agents in food and health supplements. In the further studies, in vivo experiments will be designed and completed, which will promote the development and use of *H. Rhamnoides* L.

## Data Availability

Not applicable.

## References

[1] Fang Z., YangBo Z., Xin Z., HaiYan L. (2019). Effect of white tea extract on LPS-induced inflammation of RAW264.7 cells. Tea Commun..

[2] ZiRao B. (2016). Preliminary Study on Anti-Inflammatory Effect and Molecular Mechanism of Triterpenoid Compounds in Ganoderma Lucidum.

[3] Yan-hua1 X., San-hu1 Z., Yu W., Ke-wen C., Ya-ting L., Na Y., Chun-yan Z., Jian-hua Z., Cheng-meng W. (2021). Study on the Active Substances and Antioxidant Activity of Different Solvent Extracts of Seabuckthorn Leaves. Food Res. Dev..

[4] Haonan Z., Na H., Qi D., Honglun W. (2021). Simultaneous determination of six triterpenic acids in Hippophae rhamnoides fruit by HPLC. West China J. Pharm..

[5] ZhiMin Q. (2017). Study on Anti-Inflammatory and Anti-Oxidative Stress Effects of Asiatic Acid on Acute Liver Injury and Its Mechanism.

[6] Jannus F. (2021). Efficient In Vitro and In Vivo Anti-Inflammatory Activity of a Diamine-PEGylated Oleanolic Acid Derivative. Int. J. Mol. Sci..

[7] Xuan L., YingChun H., FangLiang Z. (2021). Research progress on the pharmacological action and mechanism of hawthoric acid. Chin. J. Mod. Med..

[8] Wanqing L., Hongxiang Z., Min X., Chenglong H., Linfen T., Jun L., Ting Z., Hong C., Jing X., Chunli L. (2021). Oleanolic Acid Improves Obesity-Related Inflammation and Insulin Resistance by Regulating Macrophages Activation. Front. Pharmacol..

[9] Mengyuan F., Wencui W., Qiuming L., Weiwei W., Yang L., Hongzhuo L., Xin Y. (2021). Asiatic acid attenuates diabetic retinopathy through TLR4/MyD88/NF-κB p65 mediated modulation of microglia polarization. Life Sci..

[10] Yan-Qiu M., He T., Xiao-Xiao L., Zhen-Yu K., Qian-Wen L., Chuan-Dong X. (2020). Synthesis and anti-tumor activity of derivatives of ring A of asiatic acid. J. Asian Nat. Prod. Res..

[11] Xingfang Y., Gang Z., Zhichao H., Shangkun T., Jianchen X., Ping S., Qian T., Haixiao L. (2020). Asiatic acid ameliorates obesity-related osteoarthritis by inhibiting myeloid differentiation protein-2. Food Funct..

[12] Jiuwei C., Lin W. (2021). Maslinic Acid Inhibits Cervical Intraepithelial Neoplasia by Suppressing Interleukin-6 and Enhancing Apoptosis in a Mouse Model. Anti-Cancer Agents Med. Chem..

[13] Jing Z., Rui W., Ruihua L., Hao Y., Hengtong F. (2021). Review of the biological activity of maslinic acid1. Curr. Drug Targets.

[14] Yong Z., XiaoKui Z., YuMei S. (2017). Maslinic acid reduces cardiomyocyte injury and apoptosis mediated by high glucose and its mechanism. Heart Mag..

[15] Cai-xia T., Lei C., Yong-ting H. (2021). Maslinic acid protects against LPS-induced inflammatory response in RAW264.7 cells by regulating phosphorylation of STST3. Food Mach..

[16] Martin-Navarro C.M., Lopez-Arencibia A., Sifaoui I. (2017). Amoebicidal activityof caffeine and maslinic acid by the induction of programmed cell death in acanthamoeba. Antimicrob. Agents Chemother..

[17] Guangren X. (2020). Inhibition of chikusetsusaponin IVa on inflammatory responses in RAW264.7 cell line via MAPK pathway. Z. Für Nat. C.

[18] XiaoYi F., Wei Z., PengLun H., WenHui C., Zheng Y. (2020). Inhibitory functions of notoginseng on LPS-induced RAW264.7 cells inflammation via iNOS-NO-NFKB signaling pathways. Drug Eval. Study.

[19] Xiong L., Ouyang K.H., Jiang Y. (2018). Chemical composition of Cyclocarya paliurus polysaccharide and inflammatory effects in lipopolysaccharide-stimulated RAW264.7 macrophage. Int. J. Biol. Macromol..

[20] ChengHua C., YaJing H. (2017). The process and mechanism of inflammation mediated by LPS. J. Henan Univ..

[21] Liu X., Xie J., Jia S. (2017). Immunomodulatory effects of an acetylated Cyclocarya paliurus polysaccharide on murine macrophages RAW264.7. Int. J. Biol. Macromol..

[22] Jin Kyu K., ChangGu H. (2020). 4-Hydroxy-7-Methoxycoumarin Inhibits Inflammation in LPS-activated RAW264.7 Macrophages by Suppressing NF-κB and MAPK Activation. Molecules.

[23] Wu H. (2017). Nuciferine Ameliorates Infammatory Responses by Inhibiting the TLR4-Mediated Pathway in LipopolysaccharideInduced Acute Lung Injury. Front. Pharmacol..

[24] Borges P.V. (2017). Protective efect of gedunin on TLR-mediated infammation by modulation of infammasome activation and cytokine production: Evidence of a multitarget compound. Pharmacol. Res..

[25] Syama H.P. (2018). Syzygium cumini seed attenuates LPS induced inflammatory response in murine macrophage cell line RAW264.7 through NF-κB translocation. J. Funct. Foods.

[26] I-Chuan Y. (2018). Antrolone, a Novel Benzoid Derived from Antrodia cinnamomea, Inhibits the LPS-Induced Inflammatory Response in RAW264.7 Macrophage Cells by Balancing the NF-κB and Nrf2 Pathways. Am. J. Chin. Med..

[27] Deng L., Feng J., Cheng-song H.E. (2018). Oleanolic Acid Inhibit Tumor Necrosis Factor Alpha-induced Inflammatory Cytokines Production of Synovial Cells and Its Mechanism. Nat. Prod. Res. Dev..

[28] Si-yun C., Zhong-wen F., Li-jun P. (2021). Mechanism of asiatic acid in alcoholic hepatitis based on network pharmacology. Chin. Bull. Pharmacol..

[29] Song-bai W., Yuan-yuan W., Li-hua Z. (2018). Mechanism of hawthorn acid reducing inflammatory response and oxidative stress in mice with acute liver injury. Int. J. Biomed. Eng..

[30] Caiyun Z. (2021). The anti-inflammatory effect of-kaur-15-en-17-al-18-oic acid on lipopolysaccharide-stimulated RAW264.7 cells associated with NF-κB and P38/MAPK pathways. J. Asian Nat. Prod. Res..

[31] Qi Z.L., Qi S.M., Ling L.F. (2016). Salidroside attenuates inflammatory response via suppressing JAK2-STAT3 pathway activation and preventing STAT3 transfer into nucleus. Int. Immunopharmacol..

[32] Ren J., Li L.X., Wang Y.E. (2019). Gambogic acid induces heme oxygenase-1 through Nrf2 signaling pathway and inhibits NFkappa B and MAPK activation to reduce inflammation in LPSactivated RAW264.7 cells. Biomed Pharm..

[33] Shan H. (2019). Procyanidin A1 Alleviates Inflammatory Response induced by LPS through NF-κB, MAPK, and Nrf2/HO-1 Pathways in RAW264.7 cells. Sci. Rep..

[34] AlSaeedi Fatma J. (2020). Mangiferin protect oxidative stress against deoxynivalenol induced damages through Nrf2 signalling pathways in endothelial cells. Clin. Exp. Pharmacol. Physiol..

[35] Cheng-Cao S., Shu-Jun L., Cui-Li Y., Rui-Lin X., Yong-Yong X., Liang W., Qian-Long Z., De-Jia L. (2015). Sulforaphane Attenuates Muscle Inflammation in Dystrophin-deficient mdx Mice via NF-E2-related Factor 2 (Nrf2)-mediated Inhibition of NF-κB Signaling Pathway. J. Biol. Chem..

[36] Balsa E., Perry E.A., Bennett C.F. (2020). Defective NADPH production in mitochondrial disease complex I causes inflammation and cell death. Nat. Commun..

[37] Deo P., Chow S.H., Han M.L. (2020). Mitochondrial dysfunction caused by outer membrane vesicles from Gram-negative bacteria activates intrinsic apoptosis and inflammation. Nat. Microbiol..

